# Upregulation of cathepsin L gene under mild cold conditions in young Japanese male adults

**DOI:** 10.1186/s40101-021-00267-9

**Published:** 2021-10-22

**Authors:** Yoshiki Yasukochi, Sora Shin, Hitoshi Wakabayashi, Takafumi Maeda

**Affiliations:** 1 Department of Human Functional Genomics, Advanced Science Research Promotion Center, Organization for the Promotion of Regional Innovation, Mie University, 1577 Kurima-machiya, Tsu, Mie 514-8507 Japan; 2 Department of Genome Analysis, Institute of Biomedical Science, Kansai Medical University, 2-5-1 Shinmachi, Hirakata, Osaka 573-1010 Japan; 3 Department of Human Science, Graduate School of Design, Kyushu University, 4-9-1 Shiobaru, Minami-ku, Fukuoka, 815-8540 Japan; 4 Faculty of Engineering, Hokkaido University, N13 W8, Kita-ku, Sapporo, Hokkaido 060-8628 Japan; 5 Department of Human Science, Faculty of Design, Kyushu University, 4-9-1 Shiobaru, Minami-ku, Fukuoka, 815-8540 Japan; 6 Physiological Anthropology Research Center, Faculty of Design, Kyushu University, 4-9-1 Shiobaru, Minami-ku, Fukuoka, 815-8540 Japan

**Keywords:** Cathepsin L, Cold stress, Differentially expressed gene, RNA-seq, Thermoregulation

## Abstract

**Background:**

Physiological thermoregulatory systems in humans have been a key factor for adaptation to local environments after their exodus from Africa, particularly, to cold environments outside Africa. Recent studies using high-throughput sequencing have identified various genes responsible for cold adaptation. However, the molecular mechanisms underlying initial thermoregulation in response to acute cold exposure remain unclear. Therefore, we investigated transcriptional profiles of six young Japanese male adults exposed to acute cold stress.

**Methods:**

In a climatic chamber, the air temperature was maintained at 28°C for 65 min and was then gradually decreased to 19°C for 70 min. Saliva samples were obtained from the subjects at 28°C before and after 19°C cold exposure and were used for RNA sequencing.

**Results:**

In the cold exposure experiment, expression levels of 14 genes were significantly changed [false discovery rate (FDR) < 0.05] although the degree of transcriptional changes was not high due to experimental conditions or blunted transcriptional reaction in saliva to cold stress. As a result, differential gene expression analyses detected the cathepsin L (*CTSL*) gene to be significantly upregulated, with FDR < 0.05 and log_2_ fold change value > 1; thus, this gene was identified as a differentially expressed gene. Given that the cathepsin L protein is related to invasion of the novel coronavirus (SARS-CoV-2), mild cold stress might alter the susceptibility to coronavirus disease-19 in humans. The gene ontology enrichment analysis for 14 genes with FDR < 0.05 suggested that immune-related molecules could be activated by mild cold stress.

**Conclusions:**

The results obtained from this study indicate that *CTSL* expression levels can be altered by acute mild cold stress.

**Supplementary Information:**

The online version contains supplementary material available at 10.1186/s40101-021-00267-9.

## Background

Anatomically modern humans emerged in Africa ~300,000 to 200,000 years ago [1]; they then dispersed worldwide from Africa many tens of thousands of years ago, but the timing remains controversial [2–6]. This migration event is called “Out-of-Africa” and these migrating human populations have genetically adapted to various local environments [7, 8]. Physiological thermoregulatory ability is one of the crucial factors for adapting to local environments, especially to cold environments outside Africa. In response to cold conditions, skin blood vessels are constricted to minimize heat loss from the human body, while metabolic heat production can compensate the heat loss occurring after vasoconstriction [9]. Humans have two main types of adipose tissue, white (WAT) and brown (BAT) adipose tissues, along with a third adipose tissue called the beige or brite adipose tissue that is present in WAT (brown-like thermogenic adipocytes). BAT is involved in thermoregulatory nonshivering thermogenesis (NST) through adrenergic stimulation [10–14]. NST is defined as “heat production due to metabolic energy transformation by processes that do not involve contraction of the skeletal muscles,” whereas shivering thermogenesis is defined as “an increase in the rate of heat production during cold exposure due to increased contractile activity of the skeletal muscles not involving voluntary movements and external work” [15]. Thermoregulatory ability can be affected by lifestyles such as exercise and seasonal acclimatization [9, 16] whereas repeated cold exposure can induce insulative cold adaptation without altered metabolic response [17].

Mutations in NST-related genes may confer resistance to cold environments. Recent studies have revealed genetic factors associated with the NST response to cold stress. The uncoupling protein 1 (*UCP1*) gene is highly expressed in BAT related to adaptive adrenergic NST [18], resulting from the uncoupling of mitochondrial respiration from ATP synthesis. Single-nucleotide polymorphisms (SNPs) or haplotypes of *UCP1* can affect adaptive responses to cold environments as well as the development of obesity through energy expenditure in human populations [19, 20] and even in Japanese individuals [21–23]. Further, Japanese individuals with the AA genotype of SNP rs1057001 in the tribbles pseudokinase 2 (*TRIB2*) gene showed relatively high expression levels of several thermogenic-related genes [24], suggesting that this genotype is adaptive to cold climates. A previous study has reported that positive natural selection may have operated on a regulatory genetic variant near the transient receptor potential cation channel subfamily M member 8 (*TRPM8*) gene in Eurasian populations for cold adaptation [25], whereas strong signals of positive selection were detected in genomic regions containing carnitine palmitoyltransferase 1A (*CPT1A*) and LDL receptor-related protein 5 (*LRP5*) genes on chromosome 11 in Northeastern Siberian populations [26].

The epigenetic modification also contributes to beige/brite adipocyte thermogenesis through the TET1 (tet methylcytosine dioxygenase 1) demethylase suppression by cold stimuli [27]. RNA sequencing (RNA-seq) and lipidomic analyses of BAT in mice exposed to cold conditions exhibited upregulation of several brown adipocyte genes related to thermogenesis; further, expression levels of genes involved in glycerolipid synthesis and fatty acid elongation were also remarkably enhanced by cold exposure [28]. RNA-seq analyses of humans showed that high *UCP1* expression in the epicardial adipose tissue possibly downregulates gene expression related to immune responses such as T cell-related processes [29]. Further, transcriptome analysis using a microarray-based assay to investigate shared molecular adaptive responses between cold acclimation (14–15°C) and exercise training in the skeletal muscles of patients with type 2 diabetes showed overlapping effects on the transcriptional profiles involved in tissue remodeling [30]. A single-nucleus RNA-seq analysis of mice and human adipose tissues revealed that adipocyte subpopulations, which were identified by “P4 cells” in the study, likely regulate the thermogenic function of other adipocytes [31]. Although many studies on cold adaptation have been reported, little is known about the effects of acute cold stress on the transcriptional profiles of humans in vivo.

As mentioned above, previous genetic studies on cold adaptation have mainly focused on genetic factors responsible for the NST in BAT. However, vasoconstriction of the skin vessels plays a key role in the initial physiological response against acute cold exposure, and this response results in suppression of heat loss from the body to cold environments. To the best of our knowledge, the molecular mechanisms involved in initial thermoregulation remain unknown. Therefore, in this study, we performed the transcriptomic analysis of young Japanese male adults who were exposed to acute mild cold stress, aiming to elucidate the molecular mechanisms underlying physiological responses to cold environments.

## Methods

### Study subjects

In total, six young Japanese males aged > 20 years (mean age ± standard deviation, 21.8 ± 1.2 years) were recruited from the subject group that participated in our previous study on thermoregulatory and circulatory responses in hypobaric hypoxia and cold [32]. The anthropometric data of the study subjects are shown in Table 1. The subjects had abstained from drinking alcohol, doing exercise, and smoking for 24 h before the experiment, and had been prohibited from drinking caffeine and eating 2 h prior to the experiment, which was conducted in a climatic chamber of the Research Center for Environmental Adaptation (Fukuoka, Japan).

**Table 1:** Physical characteristics of six Japanese male subjects

**Characteristic**	**Mean ± SD**
Age (years)	21.8 ± 1.2
Height (cm)	176.0 ± 6.0
Weight (kg)	65.0 ± 8.6
Body mass index (kg/m^2^)	21.0 ± 3.1
Body fat (%)	16.5 ± 6.1
Body surface area (m^2^)	1.5 ± 0.1
Muscle percentage (%)	35.9 ± 2.6

*SD* standard deviation

### Experimental design

The cold exposure experiment was carried out from October to December 2018. The study subjects who wore undershorts and short-sleeve T-shirts (~0.13 clo) participated in the experiment. The total duration of the experiment was 135 min, and the subjects rested in a supine position during the experiment. The air temperature (T_air_) was maintained at 28°C for 65 min, gradually decreased to 19°C for 70 min, and then returned to 28°C for 30 min. Humidity was maintained at 50% RH (relative humidity) during the experiment. For transcriptome analyses, we collected the subjects’ saliva with a volume ≥ 2 mL using the Oragene® RNA kit (DNA Genotek, Ottawa, Canada) at 28°C before and after 19°C cold exposure.

### RNA sequencing and differential gene expression analyses

We extracted total RNA from saliva samples, which contain buccal cells and blood leukocytes, using either the RNeasy micro kit (Qiagen, Hilden, Germany) or the NucleoSpin® RNA kit (Takara Bio, Shiga, Japan) according to the respective manufacturers’ protocols. The RNA quality for each sample is shown in Additional file 1: Table S1. The sequence library was prepared using the TruSeq® Stranded Total RNA Library Prep kit (Illumina, San Diego, CA, USA) after RNA integrity was determined using an Agilent 2100 Bioanalyzer or TapeStation (Agilent Technologies, Santa Clara, CA, USA). RNA-seq was conducted on an Illumina NovaSeq 6000 platform (Illumina) with 100-bp paired-end (PE) reads. The quality check, library preparation, and RNA-seq were performed by Macrogen (Seoul, South Korea). We removed the Illumina PE adapters and low-quality reads using fastp ver. 0.19.7 [33]. PE reads were mapped to the human reference genome (GRCh38) using STAR ver. 2.5.3 [34]. We estimated the gene expression levels from the mapped BAM (binary alignment map) files using RSEM ver. 1.3.0 [35].

Based on the read-count data at the gene level generated using the RSEM, genes with < 13 read counts across all samples were filtered out for further analysis. Normalization of count data and estimation of log_2_ fold change (LFC) between the experimental conditions were performed using the Bioconductor R package DESeq2 ver. 1.22.2 [36] via R software ver. 3.5.3 [37] in RStudio ver. 1.2.5019 [38]. The *p* value for the significance of gene expression differences was adjusted to the false discovery rate (FDR) using the Benjamini and Hochberg method [39] for multiple testing. The differentially expressed gene (DEG) with an FDR < 0.05 and |LFC| ≥ 1 was identified. We conducted principal component analysis (PCA) to compare transcriptomic similarity among samples. The detailed methods mentioned above have been described previously [40]. The molecular function of DEG was predicted using the information on the AmiGO 2 (http://amigo.geneontology.org/amigo, [41]) and Kyoto Encyclopedia of Genes and Genomes (KEGG; https://www.genome.jp/kegg/) databases. Gene Ontology (GO) enrichment analysis was carried out using 11 upregulated and 3 downregulated genes with FDR < 0.05 in the Bioconductor R package topGO ver. 2.34.0 [42, 43].

### Real-time reverse transcription quantitative PCR analysis

To confirm the expression levels of DEG, we performed real-time reverse transcription quantitative PCR (real-time RT-qPCR) analysis. The total RNA was isolated from the saliva samples mentioned above, according to combined protocols of the Oragene® RNA kit and mirVana™ miRNA isolation kit (Ambion, Austin, TX, USA) without adding the Lysis/Binding solution and miRNA Homogenate Additive [44, 45]. The quality of total RNA was evaluated using a Nanodrop ND-1000 Spectrophotometer (Thermo Fisher Scientific, Waltham, MA, USA) (Additional file 1: Table S1). Two saliva samples from an individual were removed for further analyses due to low sample volume.

Complementary DNA (cDNA) was synthesized from total RNA by reverse transcription with oligo(dT)_20_ primers, which are useful for excluding bacterial RNA in saliva samples [45], using the Superscript III First-Strand Synthesis System (Thermo Fisher Scientific). The experimental procedure was conducted according to the manufacturer’s protocol. Real-time RT-qPCR with two technical replicates for each of five biological replicates was performed using Applied Biosystems™ TaqMan® Fast Advanced Master Mix (Thermo Fisher Scientific) and gene-specific primer probes for the cathepsin L (*CTSL*, Hs00964650_m1) and glyceraldehyde-3-phosphate dehydrogenase (*GAPDH*, Hs03929097_g1) genes, according to the manufacturer’s protocols. For real-time RT-qPCR, Thermal Cycler Dice® Real-Time System Lite TP760 (Takara Bio) was used with the following cycling conditions; 2 min at 50 °C, 20 s at 95 °C, and 50 cycles of 3 s at 95 °C and 30 s at 60 °C. Relative gene expression levels were determined using the comparative Ct (2^-ΔΔCt^) method [46] with the second derivative maximum method. The expression levels of the target gene were normalized to those of *GAPDH*.

## Results and discussion

### RNA-seq data of 12 saliva samples

**Fig. 1:**
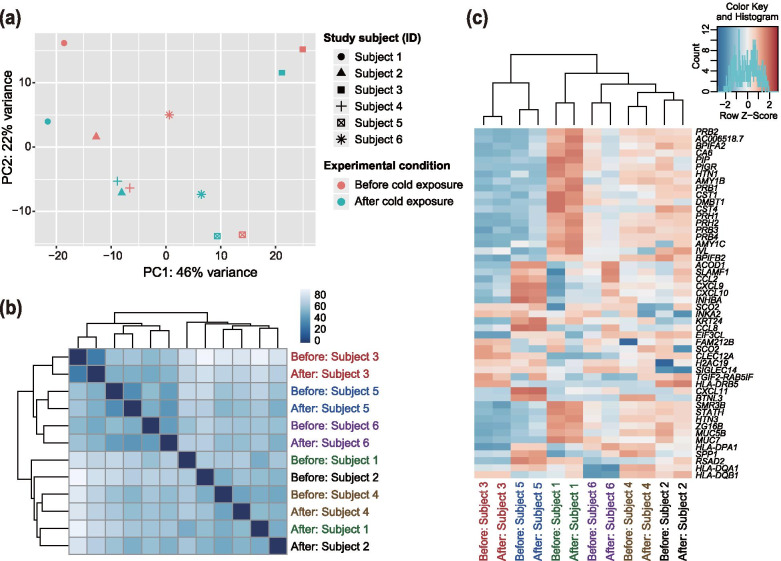
Transcriptomic similarities among 12 samples from six Japanese participants in the cold exposure experiments. **a** Principal component analysis of RNA-seq data plotted according to the first (horizontal axis) and second (vertical axis) principal components. **b** Heatmap of Euclidean distance between expression vectors. Euclidean distance was calculated using the R dist function. The color intensity reflects the transcriptomic similarity between samples. **c** Hierarchical clustering of expression for 50 genes with the highest variance across samples. Colors represent normalized gene expression levels

We obtained twelve saliva samples from six study subjects to examine the transcriptomic changes after mild cold exposure. RNA-seq analysis for saliva samples generated a total of ~562 million reads (46.8 ± 5.4 million reads). After quality control using the fastp, 20.9 ± 2.8 million reads (16.3–27.3 million reads) were uniquely mapped to the human reference genome assembly. PCA (Fig. 1a) and Euclidean distance analysis (Fig. 1b) indicated that the expression vectors of samples from the same individuals tended to be clustered, irrespective of experimental conditions. This result is similar to that of our previous study on transcriptomic changes in response to hypobaric hypoxic exposure [40]. The transcriptomic profiles of 50 genes with the highest variance across samples also showed the clustering of vectors from each individual (Fig. 1c). These results indicate that transcriptional fluctuations in response to mild cold exposure at T_air_ of 19°C were limited within each individual.

The limited expression levels may be attributed to the following: (1) the intensity or duration of cold stress may have been insufficient to induce drastic transcriptional changes, (2) saliva samples containing buccal cells and leukocytes were less sensitive to cold exposure, (3) the timing of sample collection possibly decreases the statistical power to detect DEGs because we investigated transcriptional profiles after cold exposure but not during the exposure, and (4) seasonal acclimatization related to the low outside temperature in winter (October to December) may have affected the sensitivity of transcriptional responses to cold exposure in this experiment. Transcriptional profiles differ between WAT and BAT in humans [47] and mice [48] during cold exposure; thus, it is possible that the transcriptional profiles of saliva samples are largely different from those of adipose tissues under cold conditions and that the transcriptional dynamics are less sensitive to cold stress. To understand the molecular mechanisms underlying physiological responses to cold exposure, further analyses for transcriptomic profiles at several time points during different intensities of cold stress across various cell lines or tissues are required.

### A differentially expressed gene in response to cold exposure

**Fig. 2:**
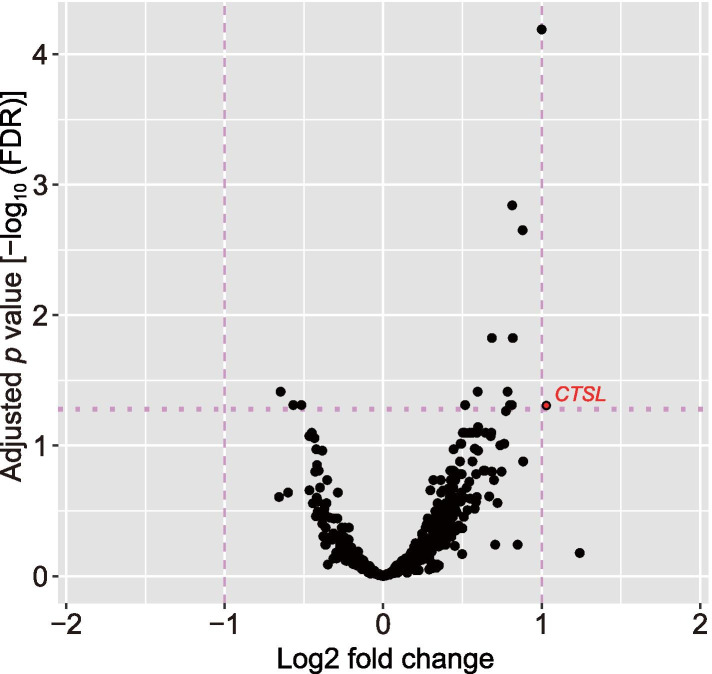
Volcano plot for differential expression analysis between two experimental conditions, 28°C before and after 19°C cold exposure. The vertical and horizontal axes represent significant difference as –log_10_(FDR) and LFC, respectively. Broken lines represent the significance or LFC threshold

To explore the significant changes in gene expression levels before and after 19°C cold stress, we conducted differential expression analysis. This analysis detected 11 and 3 significantly (FDR < 0.05) upregulated and downregulated genes, respectively (Table 2). However, the |LFC| value of 13 genes was less than unity (equivalently, their expression levels after cold exposure were less than twice compared to those before the exposure), whereas the value of the cathepsin L (*CTSL*) gene alone was higher than unity (Fig. 2; LFC = 1.03, FDR = 0.049). Therefore, we identified *CTSL* as the DEG in the present study. Although the remaining genes did not reach the |LFC| threshold, some of the genes with smaller changes had sufficient read counts compared with *CTSL*, and these changes seemed to be sufficiently reliable. Therefore, we identified the 13 genes as moderate DEGs. Differential expression analysis for the hypobaric hypoxic experiment in our previous study identified 30 upregulated DEGs [40], whereas the present study on cold exposure detected a single upregulated DEG (i.e., *CTSL*). The sample size of the previous study (three individuals) was smaller than that of the present study (six individuals); additionally, we confirmed no significant difference in the gene expression levels under no-stimulus conditions [40]. Consequently, the small number of the DEGs observed in the present study is unlikely due to the samples size and might be attributed to the possibilities described above (i.e., mild cold stress, short-term cold exposure, blunted transcriptional response in saliva to cold stress, or seasonal acclimatization).

**Table 2:** Fourteen protein coding genes with FDR < 0.05 in the differential gene expression analysis

**Expression**	**Gene symbol**	**Full gene name**	**LFC**	**Wald statistic**	***P* value**	**FDR**
Upregulation	*CD177*	CD177 molecule	0.9996	5.44	5.29 × 10^-8^	6.46 × 10^-5^
	*KLK13*	Kallikrein-related peptidase 13	0.8138	4.72	2.36 × 10^-6^	0.0014
	*S100A7*	S100 calcium-binding protein A7	0.8795	4.55	5.48 × 10^-6^	0.0022
	*ALOX5AP*	Arachidonate 5-lipoxygenase-activating protein	0.6857	4.01	6.12 × 10^-5^	0.0150
	*UPP1*	Uridine phosphorylase 1	0.8176	4.02	5.73 × 10^-5^	0.0150
	*KLK6*	Kallikrein-related peptidase 6	0.7845	3.66	2.53 × 10^-4^	0.0386
	*SPRR1B*	Small proline-rich protein 1B	0.5961	3.67	2.46 × 10^-4^	0.0386
	*TIMP1*	TIMP metallopeptidase inhibitor 1	0.8114	3.55	3.82 × 10^-4^	0.0488
	*CTSL*	Cathepsin L	1.0320	3.48	5.05 × 10^-4^	0.0488
	*C15orf48*	Chromosome 15 open-reading frame 48	0.5169	3.53	4.13 × 10^-4^	0.0488
	*GRINA*	Glutamate ionotropic receptor NMDA type subunit-associated protein 1	0.8003	3.51	4.42 × 10^-4^	0.0488
Downregulation	*PLEKHG3*	Pleckstrin homology and RhoGEF domain containing G3	−0.6475	−3.66	2.52 × 10^-4^	0.0386
	*EGR3*	Early growth response 3	−0.5158	−3.45	5.59 × 10^-4^	0.0488
	*TXNIP*	Thioredoxin interacting protein	−0.5670	−3.45	5.55 × 10^-4^	0.0488

*FDR* False discovery rate, *LFC* Log_2_ fold change

**Fig. 3:**
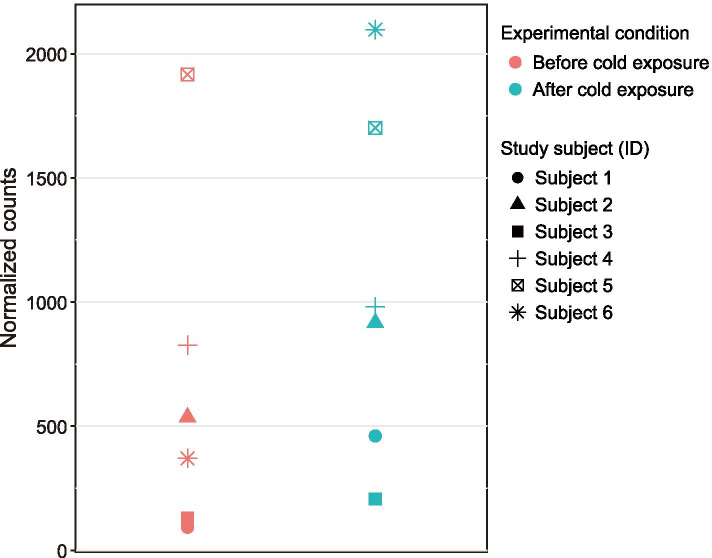
Gene expression levels of *CTSL* with LFC = 1.03, identified using the DESeq2 program, in each of the study subjects. Normalized counts at the vertical axis represent the estimated expression abundance at the gene level

Next, we compared the expression levels of *CTSL* in each individual before and after cold exposure (Fig. 3). The comparative analysis indicated that the magnitude of gene expression changes after cold exposure varied with individuals. It would be intriguing to survey the genetic variants that impact the expression levels of *CTSL* in Japanese individuals. Promoter or enhancer regions are more likely to be the factor affecting transcriptional variations; however, we did not collect DNA samples. It will thus be desirable to examine genetic variants in the *CTSL* genomic region, including neighboring regions, to detect the genetic factors related to the differences in the transcriptional changes. The expression levels of the other 13 genes with FDR < 0.05 are shown in Additional file 2: Fig. S1. As with the *CTSL*, the interindividual variability of transcriptome profiles was observed across the genes.

To confirm the difference in the expression levels of *CTSL* between the two experimental conditions (before and after 19°C cold exposure), a relative quantification analysis was conducted using the 2^-ΔΔCt^ method after real-time RT-qPCR using saliva samples from five individuals. The mean values of relative quantification in the study subjects were higher after cold exposure than before the exposure with a mean fold change of 2.87 (Additional file 3: Fig. S2), suggesting that the expression levels of *CTSL* were upregulated after acute cold exposure. However, the difference in the mean values of relative quantification was not statistically significant (*p* = 0.44, by Wilcoxon signed-rank test). It is possible that the salivary RNA used for the RT-qPCR was degraded because this experiment was conducted at least 1 year later after the RNA-seq analysis. In fact, the gene expression level of subject 4 before cold exposure appeared to be curiously higher than those of other subjects (Additional file 3: Fig S2). This may result from the low quality of RNA samples, even though the transcriptional data were normalized. The results of RT-qPCR analysis showed similar trends to those of RNA-seq analysis; however, further analysis of real-time RT-qPCR with a larger sample size is required in the future.

### Gene ontology enrichment analysis

To estimate which biological process is activated by cold stimuli, we performed topGO enrichment analysis using the DEG and moderate DEGs. The hierarchical tree of GO terms is shown in Additional file 3: Fig. S3. The enrichment analysis identified a total of eight significantly overrepresented GO terms in the biological process category (FDR < 0.05, by Fisher’s exact test). All the GO terms were related to migrations of white blood cell subtypes such as leukocyte migration (Table 3); thus, molecules involved in immune functions may be activated by the cold exposure experiments. In fact, it has been reported that acute mild cold stress may affect the proliferation of several white blood cell subtypes although effects of the proliferation on host defense remain unknown [49]. Incidentally, the expression change in the CD177 molecule (*CD177*) gene was the most statistically significant (FDR = 6.46×10^-5^) in the differential expression analysis. CD177 can be a neutrophil activation marker, and it has been reported that the expression levels were positively correlated with the severity of coronavirus disease-19 (COVID-19) [50]. Since the LFC value of *CD177* (LFC = 0.999) was quite close to the threshold (LFC = 1.000), it is possible that *CD177* is upregulated by cold stress. We will examine the transcriptional alteration of this gene under improved experimental conditions in the future.

**Table 3:** GO processes estimated by topGO enrichment analysis using 11 upregulated and 3 downregulated genes with FDR < 0.05

**GO ID**	**FDR**	**Description**
GO:0097530	3.60×10^-5^	Granulocyte migration
GO:0050900	3.60×10^-5^	Leukocyte migration
GO:0002684	6.10×10^-5^	Positive regulation of immune system process
GO:0097529	0.00011	Myeloid leukocyte migration
GO:0042742	0.00012	Defense response to bacterium
GO:2000406	0.00013	Positive regulation of T cell migration
GO:2000403	0.00019	Positive regulation of lymphocyte migration
GO:2000404	0.00021	Regulation of T cell migration

*GO* Gene ontology, *FDR* False discovery rate

### Functional prediction of the cathepsin L gene

The cathepsin L protein is a lysosomal cysteine proteinase involved in the catabolism of intracellular proteins such as collagen and elastin [51, 52]. The GO terms of the human *CTSL* included fibronectin binding, Toll-like receptor signaling pathway, adaptive immune response, cysteine-type endopeptidase activity, and protein binding. According to the KEGG database, *CTSL* plays major roles in immune responses including antigen processing and presentation (Table 4). The cathepsin L protein has various functional activities including degradation of cellular proteins and autophagic degradation [53].

**Table 4:** Kyoto encyclopedia of genes and genomes (KEGG) pathway of *CTSL*

**KEGG ID**	**Pathway**
hsa05205	Proteoglycans in cancer
hsa04210	Apoptosis
hsa05323	Rheumatoid arthritis
hsa04145	Phagosome
hsa05418	Fluid shear stress and atherosclerosis
hsa04142	Lysosome
hsa04140	Autophagy
hsa04612	Antigen processing and presentation

Notably, the cathepsin L protein is reported to be involved in cleaving the spike protein of severe acute respiratory syndrome coronavirus 2 (SARS-CoV-2) [54–56]. The inhibition of cathepsin L can decrease viral infectivity, such as herpes simplex virus 2 [57]. In addition, the cathepsin L protein is required for SARS-CoV-2 invasion into the host cell [54, 56] and cold conditions may be a potential risk factor in the spread of COVID-19 [58]. One may hypothesize that cold stress increases susceptibility to COVID-19 infection in humans via the transcriptional changes in *CTSL*. Further analyses for transcriptional changes in response to different intensities of cold stress with different sampling points are required to examine whether consistent *CTSL* expression patterns are observed.

The present study had several limitations. First, the transcriptional profiles observed in the present study were derived from saliva samples only; thus, transcriptome analyses of other tissues such as skin and blood are required because the gene expression patterns among tissues are different. Second, the effects of middle- or long-term acclimatization to cold stress remain unclear. Third, as the significant gene expression change in *CTSL* was not replicated, a replication study in other Japanese cohorts is required to validate this expression change. Fourth, the functional relevance of *CTSL* upregulation to physiological changes in response to cold stress remains unclear and needs to be elucidated.

## Conclusions

In conclusion, relatively mild cold stress did not greatly alter the transcriptional profiles of saliva samples from the study subjects, but the expression level of *CTSL* was significantly upregulated. This upregulation might be related to the immune responses induced by short-term cold stress. |LFC| values of 13 moderate DEGs were less than unity; however, transcriptional changes in some of the moderate DEGs might be involved in the physiological response to cold stress because some of these moderate DEGs showed a sufficient significant difference in transcriptional profiles compared with *CTSL*.

## Supplementary Information

**Additional file 1: Table S1.** The quality of total RNA used in RNA-seq and real-time RT-qPCR analyses.

**Additional file 2: Fig. S1.** Gene expression levels of 13 differentially expressed genes with |LFC| < 1, identified using the DESeq2 program, in each of the study subjects. Normalized counts at the vertical axis represent the estimated expression abundance at the gene level.

**Additional file 3: Fig. S2.** Relative quantification levels of *CTSL* measured using real-time RT-qPCR and the comparative Ct method. The bold black bars represent median values. The vertical axis represents the mean relative quantification of *CTSL* transcripts.

**Additional file 4: Fig. S3.** Hierarchical tree of GO terms in the biological process category. Significantly overrepresented GO terms (FDR < 0.05) are shown in red characters.

## Data Availability

The datasets presented in this article are not readily available because of ethical and privacy issues. However, scientifically motivated requests for data sharing will be considered by the reviewing ethical committee of our institute.

## Abbreviations

BAM: Binary alignment map
BAT: Brown adipose tissue
cDNA: Complementary DNA
COVID-19: Coronavirus disease-19
CPT1A: Carnitine palmitoyltransferase 1A
CTSL: Cathepsin L
DEG: Differentially expressed gene
FDR: False discovery rate
GAPDH: Glyceraldehyde-3-phosphate dehydrogenase
GO: Gene ontology
KEGG: Kyoto Encyclopedia of Genes and Genomes
LFC: Log_2_ fold change
LRP5: LDL receptor-related protein 5
NST: Nonshivering thermogenesis
PCA: Principal component analysis
PE: Paired-end
RH: Relative humidity
RNA-seq: RNA sequencing
RT-qPCR: Reverse transcription quantitative PCR
SARS-CoV-2: Severe acute respiratory syndrome coronavirus 2
SNP: Single-nucleotide polymorphism
T_air_: Air temperature
TET1: Tet methylcytosine dioxygenase 1
TRIB2: Tribbles pseudokinase 2
TRPM8: Transient receptor potential cation channel subfamily M member 8
UCP1: Uncoupling protein 1
WAT: White adipose tissue
