# Supplementary material for: Upregulation of cathepsin L gene under mild cold conditions in young Japanese male adults
Source: J Physiol Anthropol. 2021 Oct 22;40:16. doi: 10.1186/s40101-021-00267-9 (PMC8533667; doi:10.1186/s40101-021-00267-9)
Supplement: Supplementary file 1 — Additional file 1: Table S1. The quality of total RNA used in RNA-seq and real-time RT-qPCR analyses. [file 40101_2021_267_MOESM1_ESM.pdf]

Table S1. The quality of total RNA used in RNA-seq and real-time RT-qPCR analyses

Study subject ID	RNA-seq		Real-time RT-qPCR	
(Experimental condition)	ng/ul	RIN	ng/ul	260/280
Subject 1 (pre-19 °C)[a]	17.75	6.00	17.50	1.76
Subject 1 (post-19 °C)[a]	33.30	6.50	15.00	1.75
Subject 2 (pre-19 °C)	31.99	6.00	42.40	1.91
Subject 2 (post-19 °C)	64.38	5.00	73.00	1.93
Subject 3 (pre-19 °C)	68.48	5.30	74.90	1.79
Subject 3 (post-19 °C)	124.37	5.30	68.30	1.80
Subject 4 (pre-19 °C)	24.01	6.10	35.00	1.96
Subject 4 (post-19 °C)	52.08	5.50	62.10	1.84
Subject 5 (pre-19 °C)	245.28	7.30	63.70	1.89
Subject 5 (post-19 °C)	128.47	7.00	55.80	1.93
Subject 6 (pre-19 °C)	33.41	6.80	35.20	2.00
Subject 6 (post-19 °C)	77.16	6.50	24.40	1.76

RT-qPCR, reverse transcription quantitative PCR. RIN, RNA Integrity Number. Pre-19 °C, before 19 °C cold exposure. Post-19 °C, after 19 °C cold exposure.

[a] Subject 1 was removed from real-time RT-qPCR analysis.
