# Supplementary material for: Upregulation of cathepsin L gene under mild cold conditions in young Japanese male adults
Source: J Physiol Anthropol. 2021 Oct 22;40:16. doi: 10.1186/s40101-021-00267-9 (PMC8533667; doi:10.1186/s40101-021-00267-9)
Supplement: Supplementary file 2 — Additional file 2: Fig. S1. Gene expression levels of 13 differentially expressed genes with |LFC| < 1, identified using the DESeq2 program, in each of the study subjects. Normalized counts at the vertical axis represent the estimated expression abundance at the gene level. [file 40101_2021_267_MOESM2_ESM.pdf]

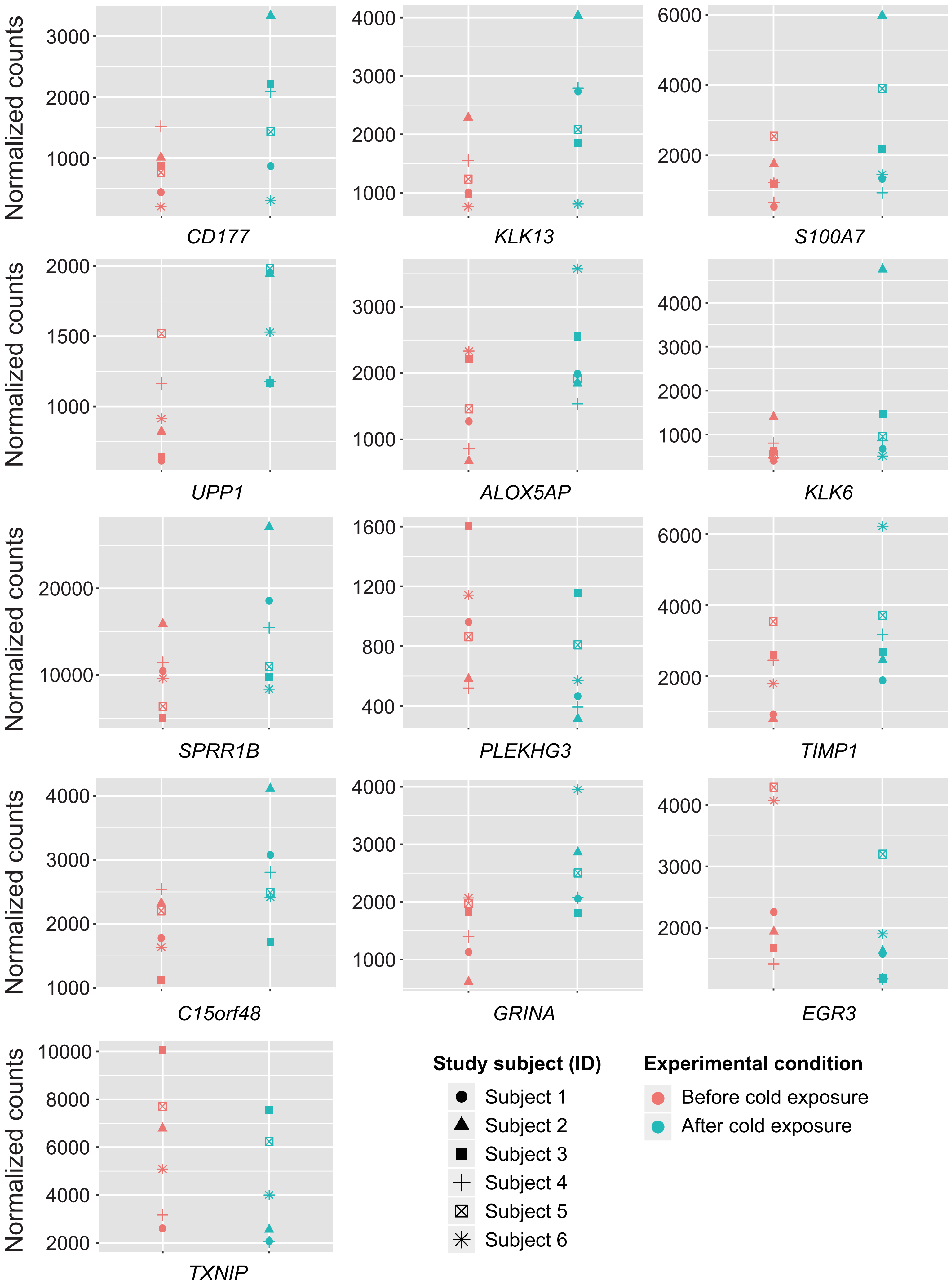

Fig. S1. Gene expression levels of 13 differentially expressed genes with |LFC| < 1, identified using the DESeq2 program, in each of the study subjects. Normalized counts at the vertical axis represent the estimated expression abundance at the gene level.
