# Supplementary material for: Upregulation of cathepsin L gene under mild cold conditions in young Japanese male adults
Source: J Physiol Anthropol. 2021 Oct 22;40:16. doi: 10.1186/s40101-021-00267-9 (PMC8533667; doi:10.1186/s40101-021-00267-9)
Supplement: Supplementary file 3 — Additional file 3: Fig. S2. Relative quantification levels of CTSL measured using real-time RT-qPCR and the comparative Ct method. The bold black bars represent median values. The vertical axis represents the mean relative quantification of CTSL transcripts. [file 40101_2021_267_MOESM3_ESM.pdf]

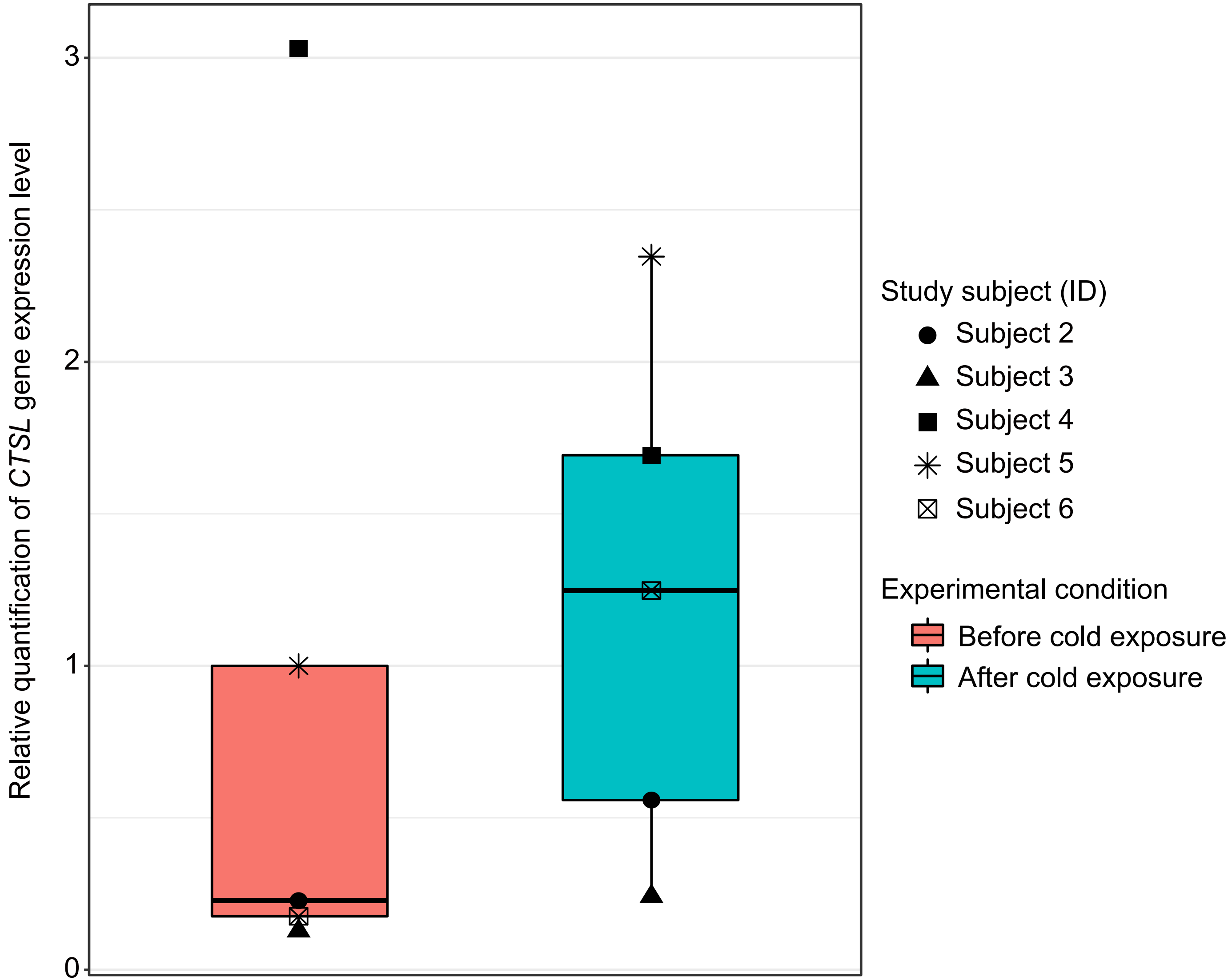

Fig. S2. Relative quantification levels of *CTSL* measured using real-time RT-qPCR and the comparative Ct method. The bold black bars represent median values. The vertical axis represents the mean relative quantification of *CTSL* transcripts.
