# Supplementary material for: Upregulation of cathepsin L gene under mild cold conditions in young Japanese male adults
Source: J Physiol Anthropol. 2021 Oct 22;40:16. doi: 10.1186/s40101-021-00267-9 (PMC8533667; doi:10.1186/s40101-021-00267-9)
Supplement: Supplementary file 4 — Additional file 4: Fig. S3. Hierarchical tree of GO terms in the biological process category. Significantly overrepresented GO terms (FDR < 0.05) are shown in red characters. [file 40101_2021_267_MOESM4_ESM.pdf]

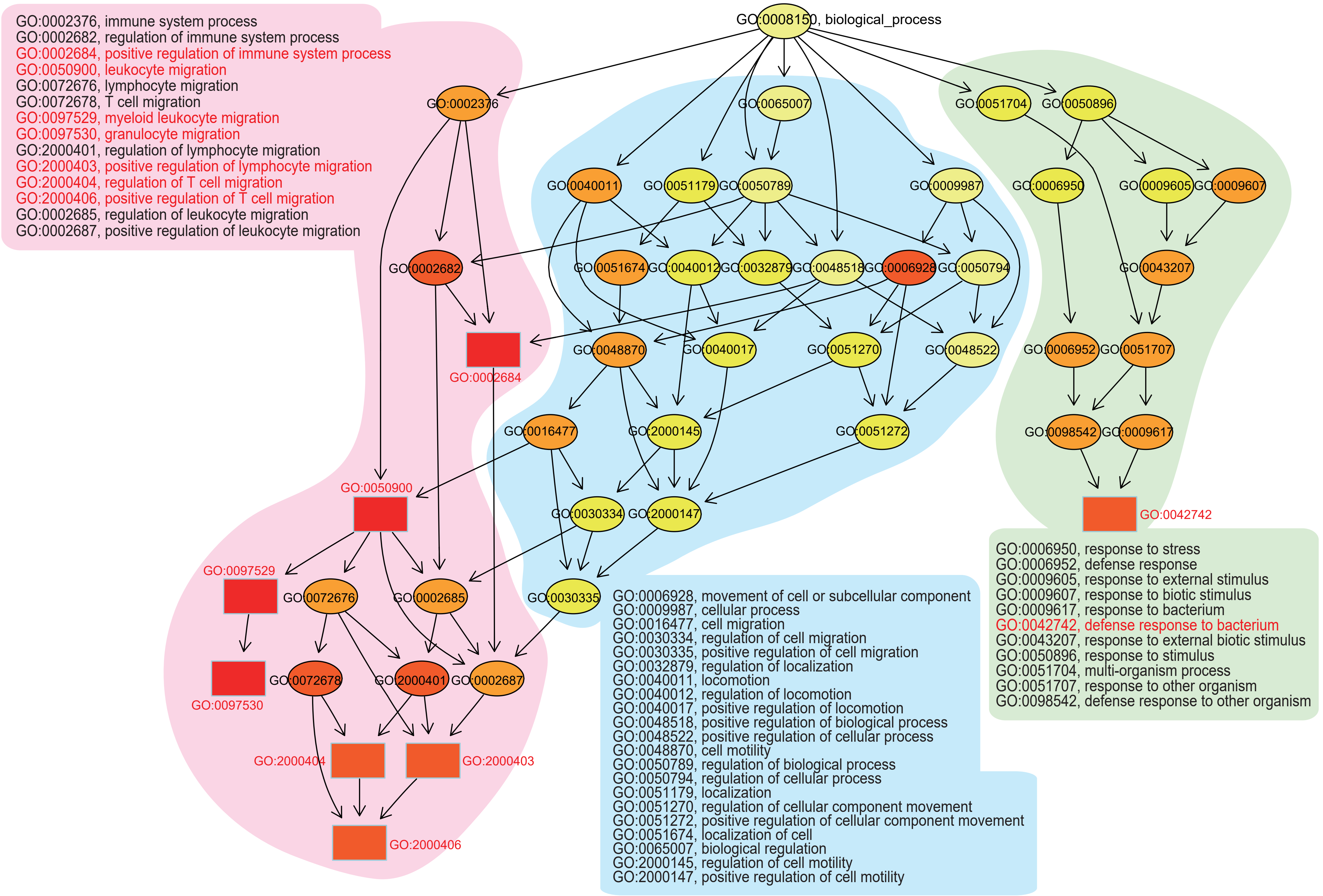

Fig. S3. Hierarchical tree of GO terms in the biological process category. Significantly overrepresented GO terms (FDR < 0.05) are shown in red characters.
